# When stroke strikes: Predictors of stroke after open thoracoabdominal aortic aneurysm repair

**DOI:** 10.1016/j.xjon.2026.102057

**Published:** 2026-08-13

**Authors:** Kyle W. Blackburn, Dominique I. Cope, Benjamin R. Zambetti, Veronica A. Glover, Susan Y. Green, Lauren K. Barron, Anna H. Xue, Vicente Orozco-Sevilla, Subhasis Chatterjee, Marc R. Moon, Joseph S. Coselli

**Affiliations:** a Division of Cardiothoracic Surgery, Michael E. DeBakey Department of Surgery, Baylor College of Medicine, Houston, Tex; b Office of Surgical Research, Michael E. DeBakey Department of Surgery, Baylor College of Medicine, Houston, Tex; c Department of Cardiothoracic Surgery, Michael E. DeBakey Department of Veterans Affairs Medical Center, Houston, Tex; d The Texas Heart Institute at Baylor College of Medicine, Houston, Tex; e Department of Cardiovascular Surgery, CHI St Luke's Health—Baylor St Luke's Medical Center, Houston, Tex; f Cardiovascular Research Institute, Baylor College of Medicine, Houston, Tex

**Keywords:** thoracoabdominal aortic aneurysm repair, stroke, atrial fibrillation, cerebrospinal fluid drainage

## Abstract

**Objective:**

Postoperative stroke after thoracoabdominal aortic aneurysm (TAAA) repair is an uncommon but devastating complication that has been understudied. This analysis aimed to identify patients at greatest risk for ischemic or hemorrhagic stroke after TAAA repair.

**Methods:**

We analyzed data from 3765 patients who underwent open TAAA repair from 1986 to 2024 at a single high-volume aortic surgery practice. We used multivariable logistic regression modeling to evaluate preoperative and operative predictors of all and persistent ischemic and hemorrhagic stroke. The effect of new-onset atrial fibrillation was evaluated.

**Results:**

Postoperative stroke persisted to death or hospital discharge in 93 patients (2.5%) and was transient in 29 (0.8%). Those with persistent stroke had a greater mortality rate (61.3%, n = 57/93) than those without any stroke (7.3%, n = 267/3643). Most persistent strokes were ischemic (76.3%) rather than hemorrhagic (26.9%). Significant predictors of persistent ischemic stroke included older age (odds ratio [OR], 1.04; *P* = .007) and chronic kidney disease (OR, 1.98; *P* = .01), whereas Crawford extent III (OR, 0.38; *P* = .01) and IV repairs (OR, 0.35; *P* = .008) were protective. Significant predictors of persistent hemorrhagic stroke included heritable thoracic aortic disease (OR, 2.34; *P* = .04) and prophylactic cerebrospinal fluid drainage (OR, 7.99; *P* = .006). Prolonged aortic clamp time (OR, 1.03, *P* = .03) was associated with greater odds of death after a stroke.

**Conclusions:**

This analysis of a large, single-practice series identified variables associated with stroke and stroke-related mortality after TAAA repair. Although stroke remains infrequent, its lethality highlights the importance of risk modeling. Mitigating the risk of hemorrhagic stroke in patients with heritable thoracic aortic disease should be prioritized.

**Figure:**
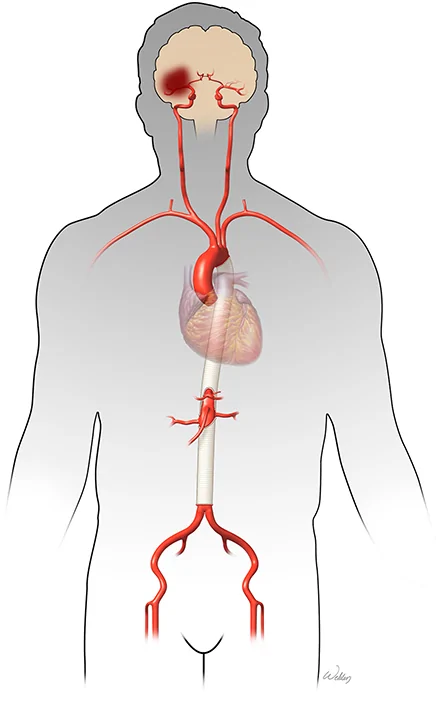
Stroke after thoracoabdominal aortic aneurysm repair.

**Central Message**

Extent I or II TAAA repair was a key predictor of ischemic stroke, whereas heritable thoracic aortic disease and prophylactic CSF drainage were key predictors of hemorrhagic stroke.

**Perspective**

Stroke after open TAAA repair is an uncommon but life-altering complication. Key contributors to ischemic stroke in patients who undergo thoracoabdominal aortic aneurysm repair included older age and extent I or II repair, with new-onset atrial fibrillation trending clinically. For hemorrhagic stroke, heritable thoracic aortic disease and prophylactic cerebrospinal fluid drainage were the main risk factors.

Patients who undergo thoracoabdominal aortic aneurysm (TAAA) repair are at high risk of postoperative complications, including stroke. Previously published rates of postoperative stroke were between 1.8% and 5.9%.^1^, ^2^, ^3^, ^4^ Furthermore, rates of postoperative atrial fibrillation (AF) have been reported at 8.5% to 23%.^5^, ^6^, ^7^ At present, predictors of stroke have not been as thoroughly explored in patients who undergo TAAA repair—particularly the effect of placing the proximal aortic clamp proximal to the left subclavian artery (LSCA) and the development of new-onset AF. Better characterizing the risk factors for postoperative stroke could aid in patient counseling and help identify patients at greatest risk for ischemic or hemorrhagic stroke after TAAA repair.

## Methods

### Study Protocol, Patient Cohort, Study Definitions, and Follow-up

Baylor College of Medicine's institutional review board approved our clinical research protocol (#18095) in February 2006, which allowed prospective capture of clinical data. Informed consent was obtained whenever possible, and a waiver of consent was approved for patients who could not give consent because they were treated before 2006 or because of illness and lack of family members to provide surrogate consent. For those treated before 2006, data were collected retrospectively from medical records.

We analyzed data from 3765 consecutive patients who underwent open TAAA repair from 1986 to 2024; data were collected using standardized definitions.^1^ Persistent strokes (PS) were symptomatic at the time of death or hospital discharge; transient strokes (TS) had symptoms that fully resolved during hospitalization. Strokes were categorized as ischemic or hemorrhagic; embolic strokes were considered ischemic. Hemorrhagic stroke was defined as any intracranial hemorrhage (eg, subdural, subarachnoid, intraparenchymal) with symptomatic neurologic sequelae. An operative death was any death within 30 days or before final hospital discharge (including any hospital transfer).^8^ Patients were considered to have heritable thoracic aortic disease (HTAD) if they had a known syndromic (eg, Marfan syndrome) or nonsyndromic genetic disorder (eg, familial thoracic aortic aneurysm and dissection) or had aortic disease at age ≤50 years. Whenever possible, postoperative surveillance occurred yearly through clinic visit, telephone interview, or written correspondence. The Social Security Death Index (up to 2011) and internet obituary searches were used to identify deaths among patients. Six patients lost to clinical follow-up at the time of hospital discharge were excluded.

### Surgical Techniques

Patients who undergo TAAA repair at our center are evaluated and optimized using the methods described by Chatterjee and colleagues.^9^ Intraoperatively, although our approach is patient-specific and nonuniform, our surgical techniques have been largely standardized since 2005.^1^ Moderate systemic heparinization (1.0-1.5 mg/kg) and mild permissive hypothermia (32-34 °C, nasopharyngeal) are used in most patients. Cold (4 °C) renal perfusion was frequently used before 2023; normothermic hyperoxygenated partial extracorporeal circulatory support was introduced after 2023 (n = 9 in this series). When applicable, segmental arteries are aggressively reattached. Prophylactic cerebrospinal fluid drainage (CSFD), left heart bypass, and selective visceral perfusion are most often used in Crawford extent II repairs.

For Crawford extents I and II, the proximal aortic clamp is commonly placed distal to the LSCA but is positioned between the left common carotid and left subclavian arteries when distal arch exposure is needed, such as in patients with chronic dissection. For extent III and IV repairs, a single clamp is typically placed in a normal segment of the aorta proximal to the aneurysm while leaving sufficient space to perform the anastomosis.

Neurologic protection involves several considerations. Preoperatively, for patients with previous stroke, a surveillance computed tomography scan is obtained preoperatively.^9^ Neurologic consultation and evaluation of functional status for pre-existing neurologic deficits are obtained if necessary. In addition, we routinely use carotid duplex ultrasonography in patients older than 60 years to evaluate concomitant carotid artery stenosis, which is treated before TAAA repair if symptomatic. For patients with HTAD, preoperative neuroimaging (magnetic resonance imaging or computed tomography of the brain) is not routine but can be obtained as needed.

Intraoperatively, we routinely use near-infrared spectroscopy for neuroperfusion monitoring. The standard practice is simple aortic crossclamping wherever possible. We reserve hypothermic circulatory arrest for complex scenarios (eg, inability to safely clamp, hemodynamic instability, atheromatous [shaggy] aorta); patients in these scenarios are at a comparably greater risk of postoperative stroke and require special attention in the postoperative period.^10^^,^^11^

For patients who develop postoperative AF, we often begin deep venous thrombosis chemoprophylaxis immediately but wait to start unfractionated heparin until postoperative day 3 and transition to oral anticoagulation on postoperative day 5 or 6. When prophylactic CSFD is used, strict volumetric protocols are followed: no more than 10 mL/h, 25 mL over any 4-hour period, or 150 mL over 24 hours.^12^ For CSFD in patients with evolving spinal cord deficit, rates up to 15 mL/h are permitted under close supervision.

For patients with suspected ischemic stroke, most are managed medically, rarely being treated with mechanical thrombectomy or catheter-directed interventions due to the often-delayed timing of discovery (eg, during early recovery and beyond the therapeutic window). For patients with suspected hemorrhagic stroke, CSFD is immediately stopped, the drains are removed, and computed tomography imaging of the head is obtained urgently; if hemorrhage is confirmed, a neurosurgical consult is immediately obtained. Anticoagulation is reversed wherever possible.

### Statistical Analysis

Statistical analyses were performed using R (version 4.4.2; The R Project for Statistical Computing). Continuous variables were presented as median [quartile 1-quartile 3] and compared by Wilcoxon rank-sum and Kruskal-Wallis tests. Categorical variables were presented as count (percentage) and compared by Pearson χ^2^ and Fisher exact tests. Survival was estimated by the Kaplan-Meier method and compared by using the log-rank test.

To control for covariates, multivariable logistic regression analyses were performed; variables were included if their univariate association with the outcome variable was significant to an alpha of 0.1. We developed 5 models. The first 4 predicted stroke: 2 primary models for ischemic and hemorrhagic PS and 2 secondary models that included both PS and TS. The fifth model predicted operative death after PS. Missing-data imputation was used for 4 variables (body mass index, maximum distal aortic diameter, chronic kidney disease, and aortic clamp time) by methods similar to those previously described.^13^

## Results

Of 3765 patients, 93 (2.5%) had PS and 29 (0.8%) had TS in the postoperative period. Of those with PS, 71 (76.3%) had an ischemic component and 22 (23.7%) had a hemorrhagic component. Of those with TS, 24 (82.8%) had an ischemic component and 5 (17.2%) had a hemorrhagic component.

### Preoperative Characteristics

The patients with PS were older than patients without stroke (*P* = .1) (Table 1) and those with TS (*P* = .3) (Table E1); those who had ischemic PS were older than those with hemorrhagic PS (*P* = .03). Although there was no difference in the rate of HTAD between patients with and without PS (*P* = .6), those who had hemorrhagic PS were more likely to have HTAD (*P* = .001) than patients with ischemic PS. Similarly, there was no difference in the rate of chronic aortic dissection between patients with and without PS (*P* = .4), but those with hemorrhagic PS more frequently had chronic aortic dissection than patients with ischemic PS (*P* = .01). Among patients with aneurysm and those with chronic dissection, ischemic PS rates were similar (1.7% vs 1.9%; *P* = .9) but hemorrhagic PS rates differed (0.4% vs 1.2%; *P* = .01). Interestingly, the ischemic and hemorrhagic PS groups did not differ significantly in the prevalence of preoperatively diagnosed AF (*P* = .5). In addition, those with PS had greater rates of most comorbidities, including cerebrovascular disease and chronic kidney disease, than those without stroke. Regarding patients with TS, Table E1 compares them with patients without stroke and those with PS; there were few differences between these groups.

**Table 1:** Preoperative characteristics of patients who underwent 3765 TAAA repairs, stratified by stroke status and type

Variable	No stroke (n = 3643)	Persistent stroke (n = 93)	*P*	Ischemic (n = 71)	Hemorrhagic (n = 22)	*P*
Age, y	67 [58-73]	70 [62-76]	.1	72 [65-77]	66 [50-71]	**.03**
Male	2272 (62.3)	60 (64.5)	.7	48 (67.6)	12 (54.5)	.3
Heritable thoracic aortic disease	623 (17.1)	14 (15.1)	.6	6 (8.5)	8 (36.4)	**.001**
Marfan syndrome	343 (9.4)	8 (8.6)	.8	3 (4.2)	5 (22.7)	**.007**
Loeys-Dietz syndrome	20 (0.5)	0	.5	0	0	–
Aortic dissection	1345 (36.9)	40 (43.0)	.2	27 (38.0)	13 (59.1)	.08
Acute or subacute	153 (4.2)	6 (6.5)	.3	6 (8.5)	0	.2
Chronic	1176 (32.3)	34 (36.6)	.4	21 (29.6)	13 (59.1)	**.01**
DeBakey type I	492 (13.5)	16 (17.2)	.3	10 (14.1)	6 (27.3)	.2
DeBakey type III	805 (22.1)	24 (25.8)	.4	17 (23.9)	7 (31.8)	.5
Localized dissection	32 (0.9)	0	.4	0	0	–
>1 dissection	105 (2.9)	5 (5.4)	.2	2 (2.8)	3 (13.6)	**.049**
Maximum distal aortic diameter, cm	6.2 [5.5-7.2] (n = 3223)	6.2 [5.5-7.0] (n = 85)	.9	6.3 [5.7-7.0] (n = 65)	5.8 [5.2-6.4] (n = 20)	.1
Hypertension	3118 (85.6)	82 (88.2)	.4	63 (88.7)	19 (86.4)	.8
Hyperlipidemia	1140 (31.3)	30 (32.3)	.9	27 (38.0)	3 (13.6)	**.03**
Diabetes	298 (8.2)	14 (15.1)	**.02**	11 (15.5)	3 (13.6)	.8
Coronary artery disease	1286 (35.3)	32 (34.4)	.9	27 (38.0)	5 (22.7)	.2
Previous CABG	552 (15.2)	14 (15.1)	>.9	11 (15.5)	3 (13.6)	.8
Previous myocardial infarction	686 (18.8)	17 (18.3)	.9	16 (22.5)	1 (4.5)	.056
Atrial fibrillation	439 (12.1)	13 (14.0)	.6	9 (12.7)	4 (18.2)	.5
Cerebrovascular disease	624 (17.1)	27 (29.0)	**.003**	21 (29.6)	6 (27.3)	.8
Previous stroke	368 (10.1)	18 (19.4)	**.004**	14 (19.7)	4 (18.2)	.9
Chronic kidney disease∗	1395 (38.3) (n = 3358)	52 (62.7) (n = 83)	**<.001**	42 (59.2) (n = 62)	10 (45.5) (n = 21)	.1
Body mass index, kg/m^2^	26 [23-29] (n = 3358)	27 [24-31] (n = 77)	.2	27 [23-29] (n = 59)	27 [23-31.5] (n = 18)	.5
Chronic obstructive pulmonary disease	1681 (46.1)	35 (37.6)	.1	29 (40.8)	6 (27.3)	.3
Tobacco use (current/past)	2835 (77.8)	74 (79.6)	.7	59 (83.1)	15 (68.2)	.1
Peripheral vascular disease	853 (23.4)	32 (34.4)	**.01**	25 (35.2)	7 (31.8)	.8
Symptomatic aneurysm	2324 (63.8)	67 (72.0)	.1	54 (76.1)	13 (59.1)	.1
Acute	618 (17.0)	17 (18.3)	.7	15 (21.1)	2 (9.1)	.2
Chronic	1809 (49.7)	52 (55.9)	.2	41 (57.7)	11 (50.0)	.5
Rupture	227 (6.2)	4 (4.3)	.4	3 (4.2)	1 (4.5)	.9
Infection	146 (4.0)	2 (2.2)	.4	2 (2.8)	0	.4
Previous open or endovascular aortic repair	1685 (46.3)	44 (47.3)	.8	32 (45.1)	12 (54.5)	.4
Previous open arch (any)	465 (12.8)	15 (16.1)	.3	10 (14.1)	5 (22.7)	.3
Previous hemiarch	204 (5.6)	5 (5.4)	.9	3 (4.2)	2 (9.1)	.4
Previous partial or total arch†	270 (7.4)	10 (10.8)	.2	7 (9.9)	3 (13.6)	.6

Values are n (%) or median [quartile 1-quartile 3]. Values in bold indicate *P* < .05. *TAAA*, Thoracoabdominal aortic aneurysm; *CABG*, coronary artery bypass grafting.

∗ Preoperative estimated glomerular filtration rate <60 mL/min/1.73 m^2^.

† Partial arch replacement included a single brachiocephalic artery in the repair; total arch replacement included at least 2 brachiocephalic arteries. Characteristics of patients with transient stroke (n = 29) are shown in Table E1.

### Operative Details

Compared with the group without stroke, the PS and TS groups had greater rates of Crawford extent II repair (*P* < .001), with associated greater rates of left heart bypass, CSFD, segmental artery reattachment, and selective visceral perfusion (Tables 2 and E2). Patients with PS had longer aortic clamp times (*P* = .02). Interestingly, the rate of aortic clamping proximal to the LSCA was not significantly different for PS patients (*P* = .6), even when the analysis was confined to extent I or II repairs or stratified by stroke type (Table E3). However, clamping proximal to the LSCA was related to TS (*P* = .002, Table E4). Hypothermic circulatory arrest was infrequently used; no significant difference in rates of PS was observed between patients who underwent hypothermic circulatory arrest versus left heart bypass (*P* = .5).

**Table 2:** Operative details of patients who underwent 3765 TAAA repairs, stratified by stroke status and type

Variable	No stroke (n = 3643)	Persistent stroke (n = 93)	*P*	Ischemic (n = 71)	Hemorrhagic (n = 22)	*P*
Elective repair	2762 (75.8)	74 (79.6)	.4	56 (78.9)	18 (81.8)	.8
Crawford extent of repair						
Extent I	956 (26.2)	25 (26.9)	.9	19 (26.8)	6 (27.3)	>.9
Extent II	1203 (33.0)	50 (53.8)	**<.001**	36 (50.7)	14 (63.6)	.3
Extent III	740 (20.3)	8 (8.6)	**.005**	8 (11.3)	0	.1
Extent IV	744 (20.4)	10 (10.8)	**.02**	8 (11.3)	2 (9.1)	.8
Aortic repair details						
Clamping proximal to LSCA	566 (15.5)	18 (19.4)	.3	13 (18.3)	5 (22.7)	.6
Distal hemiarch or other arch repair	26 (0.7)	1 (1.1)	.7	1 (1.4)	0	.6
LSCA bypass at time of repair	66 (1.8)	6 (6.5)	**.001**	4 (5.6)	2 (9.1)	.6
ET/FET completion anastomosis	180 (4.9)	9 (9.7)	**.04**	6 (8.5)	3 (13.6)	.5
Chronic untreated arch dissection	256 (7.0)	7 (7.5)	.9	5 (7.0)	2 (9.1)	.8
Segmental artery reattachment	1842 (50.6)	62 (66.7)	**.002**	44 (62.0)	18 (81.8)	.08
Endograft explantation	104 (2.9)	3 (3.2)	.8	2 (2.8)	1 (4.5)	.7
Aortic clamp time, min	51 [38-67] (n = 3625)	59 [45-74] (n = 91)	**.03**	57 [44-71] (n = 70)	64 [47-77] (n = 21)	.6
Renovisceral artery management						
Endarterectomy, stenting, or bypass grafting	1601 (43.9)	41 (44.1)	>.9	35 (49.3)	6 (27.3)	.07
Endarterectomy	941 (25.8)	24 (25.8)	>.9	23 (32.4)	1 (4.5)	**.009**
Stenting	301 (8.3)	12 (12.9)	.1	11 (15.5)	1 (4.5)	.2
Bypass grafting	1042 (28.6)	32 (34.4)	.2	26 (36.6)	6 (27.3)	.4
Adjuncts						
Cerebrospinal fluid drainage	1755 (48.2)	62 (66.7)	**<.001**	42 (59.2)	20 (90.9)	**.006**
Left heart bypass	1637 (44.9)	57 (61.3)	**.002**	41 (57.7)	16 (72.7)	.2
Hypothermic circulatory arrest	62 (1.7)	3 (3.2)	.3	2 (2.8)	1 (4.5)	.7
Cold renal perfusion	2177 (59.8)	49 (52.7)	.2	41 (57.7)	8 (36.4)	.08
Selective visceral perfusion	894 (24.5)	36 (38.7)	**.002**	23 (32.4)	13 (59.1)	**.03**

Values are n (%) or median [quartile 1-quartile 3]. Characteristics of patients with transient stroke (n = 29) are shown in Table E4. Values in bold indicate *P* < .05. *TAAA*, Thoracoabdominal aortic aneurysm; *LSCA*, left subclavian artery; *ET*, elephant trunk; *FET*, frozen elephant trunk.

### Early Outcomes

Of the 93 patients who had PS, 57 had an operative death (61.3%; *P* < .001) (Table 3). Patients with PS had greater rates of respiratory failure necessitating tracheostomy (*P* < .001) and new-onset AF (*P* = .04) than those without stroke. Operative mortality was not significantly different between patients who had TS (Table E5) and patients without stroke (*P* = .7).

**Table 3:** Early outcomes of patients who underwent 3765 TAAA repairs, stratified by stroke status and type

Variable	No stroke (n = 3643)	Persistent stroke (n = 93)	*P*	Ischemic (n = 71)	Hemorrhagic (n = 22)	*P*
Operative death	267 (7.3)	57 (61.3)	**<.001**	40 (56.3)	17 (77.3)	.08
30-d	166 (4.6)	45 (48.4)	**<.001**	31 (43.7)	14 (63.6)	.1
Spinal cord deficit	350 (9.6)	12 (12.9)	.3	10 (14.1)	2 (9.1)	.5
Persistent paraplegia∗	101 (2.8)	9 (9.7)	**<.001**	7 (9.9)	2 (9.1)	.9
Rescue cerebrospinal fluid drainage	45 (1.2)	1 (1.1)	.9	0	1 (4.5)	.07
New-onset atrial fibrillation	570 (15.6)	25 (26.9)	**.04**	21 (29.6)	4 (18.2)	.3
Respiratory failure necessitating tracheostomy	303 (8.3)	29 (31.2)	**<.001**	22 (31.0)	7 (31.8)	.9
Operative survivors						
Discharge with stroke	0	36 (38.7)	**<.001**	31 (43.7)	5 (22.7)	.08

Values are n (%).Values in bold indicate *P* < .05. *TAAA*, Thoracoabdominal aortic aneurysm.

∗ Persisting at the time of death or hospital discharge/transfer. Select outcomes of patients with transient stroke (n = 29) are shown in Table E5.

### Late Outcomes

The Kaplan-Meier analyses found that patients with stroke had poorer overall survival than patients without stroke (Figure 1). Causes of death are reported in Tables E6 and E7.

**Figure 1:**
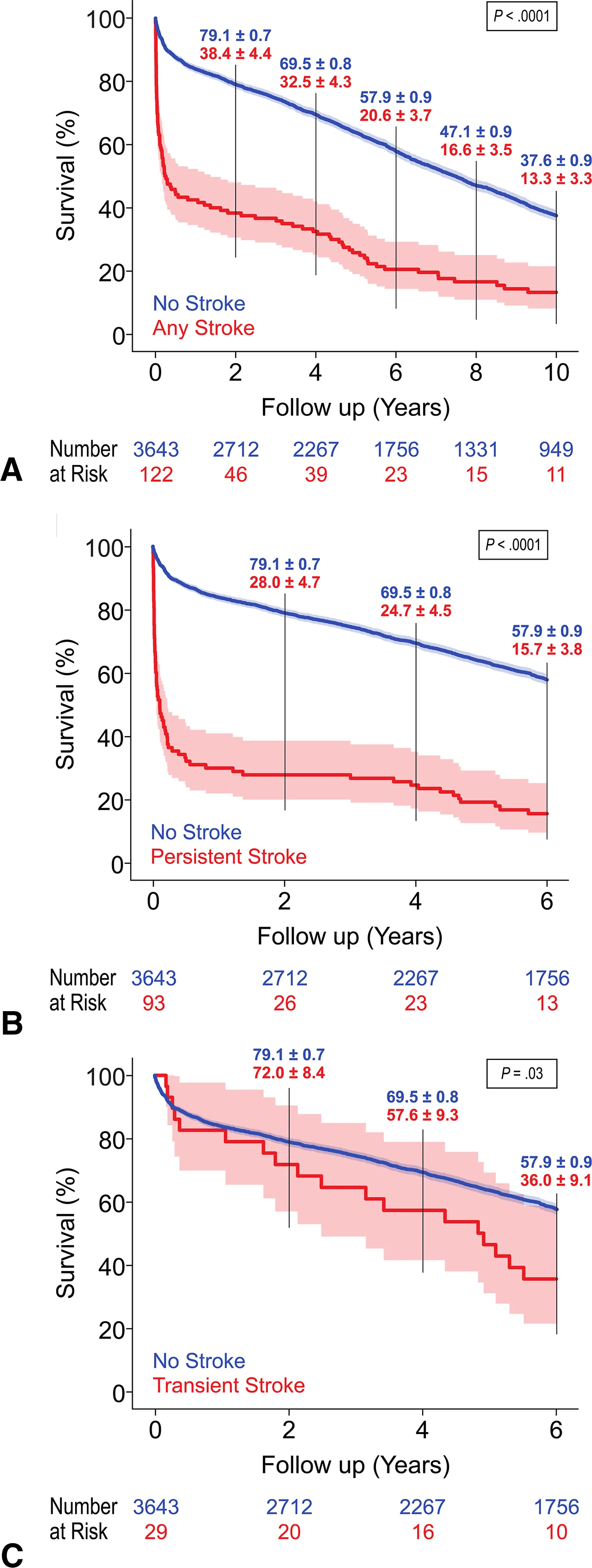
Kaplan-Meier analysis was used to compare patients who did not have a stroke (n = 3643) with patients who had (A) any stroke (persistent or transient; n = 122), (B) persistent stroke (n = 93), and (C) transient stroke (n = 29). Survival after thoracoabdominal aortic aneurysm repair was significantly poorer in patients who had postoperative stroke. 95% CI bands are shown.

### Findings From Modeling

The 4 multivariable logistic regression models built can be found in Table 4, and the associated univariate analyses are presented in Table E10, Table E11, Table E8, Table E9. Imputed data are shown in Table E12. Considering ischemic stroke (PS and TS), we identified 7 significant predictors, including older age (odds ratio [OR], 1.04; *P* = .001) and cerebrovascular disease (OR, 1.80; *P* = .01) as risk factors and Crawford extent III (OR, 0.37; *P* = .004) and IV (OR, 0.29; *P* = .001) repairs as protective factors. In addition, clamping proximal to the LSCA and new-onset AF trended toward being significant risk factors for PS and TS combined (OR, 1.58, *P* = .08) and for PS alone (OR, 1.58, *P* = .1). The risk factors for hemorrhagic PS were HTAD (OR, 2.34; *P* = .04), CSFD (OR, 7.99; *P* = .006), and selective visceral perfusion (OR, 2.71; *P* = .02); secondary model findings (PS and TS) were similar. When analysis was limited to patients with PS (n = 93), only longer aortic clamp time was associated with greater odds of operative death (OR, 1.03/min, *P* = .03) (Table E13).

**Table 4:** Details of the 4 multivariable logistic regression models built to predict stroke, stratified by stroke persistence and stroke type

Characteristics	Persistent stroke only		Transient and persistent stroke	
	OR	*P*	OR	*P*
Ischemic stroke				
Age, increasing by year	1.04	**.007**	1.04	**.001**
Cerebrovascular disease	1.67	.06	1.80	**.01**
Chronic kidney disease	1.98	**.01**	2.19	**<.001**
Body mass index, increasing by kg/m^2^	1.06	**.03**	–	–
Symptomatic aneurysm	1.86	**.03**	1.89	**.01**
Peripheral vascular disease	1.55	.1	–	–
Crawford extent III repair	0.38	**.01**	0.37	**.004**
Crawford extent IV repair	0.35	**.008**	0.29	**.001**
Clamping proximal to LSCA	–	–	1.58	.08
Aortic clamp time, increasing by minute	1.01	.06	1.01	**.01**
New-onset atrial fibrillation	1.58	.1	–	–
Hemorrhagic stroke				
Heritable thoracic aortic disease	2.34	**.04**	2.00	**.07**
Cerebrospinal fluid drainage	7.99	**.006**	6.14	**.001**
Cold renal perfusion	–	–	0.34	**.005**
Selective visceral perfusion	2.71	**.02**	2.93	**.005**

Full details of the individual models are provided in Table E10, Table E11, Table E8, Table E9. Values in bold indicate *P* < .05. *OR*, Odds ratio; *LSCA*, left subclavian artery.

## Discussion

In this study, we sought to identify patients at greatest risk for stroke after TAAA repair to inform targeted perioperative strategies aimed at improving outcomes. For ischemic PS, older age, a larger burden of comorbidities, and new-onset AF were clinical risk factors, whereas Crawford extent III and IV repairs were protective factors. For hemorrhagic PS, HTAD and use of CSFD were key contributors. For those who experienced PS after TAAA repair, longer aortic clamp time was positively associated with risk of operative death.

One result of interest was that clamping proximal to the LSCA was not a significant predictor of ischemic PS, even when only extent I or II TAAA repairs were analyzed. Some have suggested that clamping closer to the left carotid artery could increase the risk of dislodging atheromatous material or calcium, leading to an embolic stroke.^14^^,^^15^ Although our previous study did identify an elevated risk of PS in these patients,^16^ we did not observe this association in our current analysis, a finding in line with a similar study from Girardi and colleagues.^17^ However, when TS and PS were analyzed together, clamping proximal to the LSCA trended toward prediction, suggesting that more proximal clamping is clinically associated with a greater rate of TS. Working under the assumption that TSs are smaller insults caused by smaller emboli, our hypothesis is that although clamping proximal to the LSCA may not increase the risk of large embolic events that drive large deficits, it poses an increased risk of smaller insults and perhaps subclinical stroke.

To that end, although the symptoms of these TS resolved before discharge, these strokes did correspond to a diminished overall survival, even though preoperative risk factors were relatively the same between those who had and did not have a TS (Table E1). The prevalence of subclinical strokes has been a major consideration point when comparing on- and off-pump coronary artery bypass grafting,^18^ with some work showing diminished ability to perform activities of daily living. Our findings in conjunction with this body of literature suggest that aortic surgery patients with TS should be handled with care and that more research is warranted to better understand the sequelae of TS.

A common complication after cardiac surgery is new-onset AF, which poses a risk of stroke.^19^ Although new-onset AF is less common in TAAA repairs than in other cardiac cases,^5^^,^^7^^,^^20^ its incidence in our cohort was nonnegligible. Furthermore, new-onset AF trended toward being a predictor of PS, suggesting these patients should be monitored more closely for stroke symptoms and, arguably, receive postoperative anticoagulation.

A few strokes occurred in patients who had extent III and IV TAAA repairs, which our experience suggests is a result of extraoperative factors. Extent III and IV repairs were generally done to treat aneurysmal aortic disease, whereas extent I and II repairs were most often for dissection. Patients with aneurysmal aortic disease tend to be older and to have a larger burden of comorbidities, leading to a higher baseline risk of stroke. We do not believe that operative factors substantially affected these patients’ outcomes.

Two primary factors were associated with greater risk of hemorrhagic PS: HTAD and use of CSFD. Patients with HTAD are at an elevated risk for bleeding due to disruptions in collagen or extracellular matrix synthesis, leading to vessel-wall fragility.^21^ Kim and colleagues^22^ found a significantly greater risk of hemorrhagic PS in patients with Marfan syndrome (OR, 1.56; *P* = .02). For this reason, extreme vigilance in the use of anticoagulation in these patients might be prudent. In addition, CSFD is a well-established risk factor for intracranial bleeding.^23^^,^^24^ This risk is attributed to a rapid or excessive reduction in intracranial pressure, which can lead to stretching and tearing of cerebral blood vessels, most often veins. For this reason, the prophylactic value of CSFD should be weighed against the potentially greater risk of hemorrhagic PS, especially in patients with HTAD.^24^

Our study has several limitations, including those inherent to any observational and single-institutional study. In addition, our database did not contain data on the severity or timing of stroke other than whether the symptoms were persistent or transient, and as a result we could not control for any of these factors in the analysis. Because we rarely use hypothermic circulatory arrest during TAAA repair, our results may not be generalizable to centers with more frequent use.^4^

## Conclusions

Stroke is a rare but morbid complication of TAAA repair. Our data suggest that ischemic PS is associated with new-onset AF as well as other predictive comorbidities. For hemorrhagic PS, HTAD and prophylactic CSFD were primary contributors. Clamping proximal to the LSCA, although not a significant predictor of ischemic PS, did contribute to ischemic TS, suggestive of a greater burden of subclinical stroke for these patients. Particular attention should be paid to patients with these risk factors in both the intraoperative and postoperative periods to prevent stroke-associated neurologic and survival deficits.

## Conflict of Interest Statement

Dr Orozco-Sevilla participates in clinical trials for Gore Medical, Cook Medical, and Terumo Aortic. Dr Chatterjee has served on advisory boards for Edwards Lifesciences, Eagle Pharmaceuticals, La Jolla Pharmaceutical Company, and Baxter Lifesciences. Dr Moon serves on advisory boards for Edwards Lifesciences. Dr Coselli consults for and participates in clinical trials for Terumo Aortic, Medtronic, Inc, and W. L. Gore & Associates and participates in clinical trials for Abbott Laboratories, Artivion, and Edwards Lifesciences. All other authors reported no conflicts of interest.

The *Journal* policy requires editors and reviewers to disclose conflicts of interest and to decline handling or reviewing manuscripts for which they may have a conflict of interest. The editors and reviewers of this article have no conflicts of interest.

## Figures and Tables

**Table E1:** Comparison of preoperative characteristics for patients with transient postoperative stroke (n = 29), those without stroke (n = 3643), and those with persistent postoperative stroke (n = 93)

Variable	No stroke (n = 3643)	Transient stroke (n = 29)	*P*	Transient stroke (n = 29)	Persistent stroke (n = 93)	*P*
Age, y, median [Q1-Q3]	67 [58-73]	69 [60-73]	.3	69 [60-73]	70 [62-76]	.4
Male	2272 (62.3)	18 (62.1)	>.99	18 (62.1)	60 (64.5)	.8
Heritable thoracic aortic disease	623 (17.1)	2 (6.9)	.1	2 (6.9)	14 (15.1)	.3
Marfan syndrome	343 (9.4)	2 (6.9)	.6	2 (6.9)	8 (8.6)	.8
Loeys-Dietz syndrome	20 (0.5)	0	.7	0	0	–
Aortic dissection	1345 (36.9)	14 (48.3)	.2	14 (48.3)	40 (43.0)	.6
Acute or subacute	153 (4.2)	3 (10.3)	.1	3 (10.3)	6 (6.5)	.5
Chronic	1176 (32.3)	11 (37.9)	.5	11 (37.9)	34 (36.6)	.9
DeBakey type I	492 (13.5)	7 (24.1)	.1	7 (24.1)	16 (17.2)	.4
DeBakey type III	805 (22.1)	7 (24.1)	.8	7 (24.1)	24 (25.8)	.9
Localized dissection	32 (0.9)	0	.6	0	0	–
>1 dissection	105 (2.9)	1 (3.4)	.9	1 (3.4)	5 (5.4)	.7
Maximum distal aortic diameter, cm, median [Q1-Q3]	6.2 [5.5-7.2] (n = 3223)	5.8 [5.5-6.8] (n = 27)	.2	5.8 [5.5-6.8] (n = 27)	6.2 [5.5-7.0] (n = 85)	.2
Hypertension	3118 (85.6)	26 (89.7)	.6	26 (89.7)	82 (89.1)	.9
Hyperlipidemia	1140 (31.3)	12 (41.4)	.3	12 (41.4)	30 (32.3)	.4
Diabetes	298 (8.2)	2 (6.9)	.8	2 (6.9)	14 (15.1)	.3
Coronary artery disease	1286 (35.3)	10 (34.5)	.9	10 (34.5)	32 (34.4)	>.99
Previous CABG	552 (15.2)	4 (13.8)	.8	4 (13.8)	14 (15.1)	.9
Previous percutaneous intervention	363 (10.0)	4 (13.8)	.5	4 (13.8)	7 (7.5)	.3
Previous myocardial infarction	686 (18.8)	8 (27.6)	.2	8 (27.6)	17 (18.3)	.3
Atrial fibrillation	439 (12.1)	4 (13.8)	.8	4 (13.8)	13 (14.0)	>.9
Cerebrovascular disease	624 (17.1)	8 (27.6)	.1	8 (27.6)	27 (29.0)	.9
Previous stroke	368 (10.1)	2 (6.9)	.6	2 (6.9)	18 (19.4)	.1
Chronic kidney disease∗	1395 (38.3) (n = 3358)	16 (55.2) (n = 27)	.2	16 (55.2) (n = 27)	52 (62.7) (n = 83)	.8
Body mass index, kg/m^2^	26 [23-29]	26 [24-28]	.8	26 [24-28]	27 [24-31]	.2
Chronic obstructive pulmonary disease	1681 (46.1)	15 (51.7)	.5	15 (51.7)	35 (37.6)	.2
Tobacco use (current or previous)	2835 (77.8)	24 (82.8)	.5	24 (82.8)	74 (79.6)	.7
Symptomatic	2324 (63.8)	22 (75.9)	.2	22 (75.9)	67 (72.0)	.7
Acute	618 (17.0)	3 (10.3)	.3	3 (10.3)	17 (18.3)	.3
Chronic	1809 (49.7)	20 (69.0)	**.04**	20 (69.0)	52 (55.9)	.2
Peripheral vascular disease	853 (23.4)	5 (17.2)	.4	5 (17.2)	32 (34.4)	.08
Rupture	227 (6.2)	1 (3.4)	.5	1 (3.4)	4 (4.3)	.8
Infection	146 (4.0)	2 (6.9)	.4	2 (6.9)	2 (2.2)	.2
Previous open/endovascular aortic repair (any segment)	1685 (46.3)	17 (58.6)	.2	17 (58.6)	44 (47.3)	.3
Previous open arch (any type)	465 (12.8)	5 (17.2)	.5	5 (17.2)	15 (16.1)	.9
Previous hemiarch	204 (5.6)	1 (3.4)	.6	1 (3.4)	5 (5.4)	.7
Previous partial or total arch†	270 (7.4)	4 (13.8)	.2	4 (13.8)	10 (10.8)	.7

Values are n (%) or median [quartile 1-quartile 3]. Values in bold indicate *P* < .05. *CABG*, Coronary artery bypass grafting.

∗ Preoperative estimated glomerular filtration rate <60 mL/min/1.73 m^2^.

† Partial arch replacement included a single brachiocephalic artery in the repair; total arch replacement included at least 2 brachiocephalic arteries.

**Table E2:** Use of cerebrospinal fluid drainage, stratified by stroke type and Crawford extent of repair

Variable	No stroke (n = 3643)	Transient stroke (n = 29)	Persistent stroke		*P*
			Ischemic stroke (n = 71)	Hemorrhagic stroke (n = 22)	
All extents of repair					
CSFD (intraoperative)	1755 (48.2)	21 (72.4)	42 (59.2)	20 (90.9)	**<.001**
CSFD (rescue)	45 (1.3)	1 (3.4)	0	1 (4.5)	.3
Crawford extent II repairs only	n = 1203	n = 12	n = 36	n = 14	
CSFD (intraoperative)	778 (64.7)	11 (91.7)	26 (72.2)	13 (92.9)	**.03**
CSFD (rescue)	23 (1.9)	0	0	0	.8
Crawford extents I and II repairs only	n = 2159	n = 26	n = 55	n = 20	
CSFD (intraoperative)	1284 (59.5)	21 (80.8)	37 (67.3)	18 (90.0)	**.003**
CSFD (rescue)	26 (1.2)	1 (3.8)	0	0	.5

Values are n (%). Values in bold indicate *P* < .05. Most commonly, CSFD is started when repair begins. Occasionally, CSFD is used postoperatively as a rescue measure, in hopes of reversing symptoms of spinal cord deficit in patients who did not previously undergo such drainage or whose catheter was recently removed. Values for patients without CSFD are not shown. *CSFD*, Cerebrospinal fluid drainage.

**Table E3:** Clamping proximal to the left subclavian artery, stratified by stroke type and extent of repair

Variable	No stroke (n = 3643)	Transient stroke (n = 29)	Persistent stroke		*P*
			Ischemic stroke (n = 71)	Hemorrhagic stroke (n = 22)	
All extents of repair					
Clamping proximal to the LSCA	566 (15.5)	11 (37.9)	13 (18.3)	5 (22.7)	**.007**
Crawford extent II repairs only	n = 1265	n = 12	n = 36	n = 14	
Clamping proximal to the LSCA	276 (22.9)	4 (33.3)	8 (22.2)	3 (21.4)	.9
Crawford extents I and II repairs only	n = 2159	n = 26	n = 55	n = 20	
Clamping proximal to the LSCA	561 (26.0)	11 (42.3)	13 (23.6)	5 (25.0)	.3

Values are n (%). Values for patients with an aortic clamp site distal to the LSCA are not shown. Values in bold indicate *P* < .05. *LSCA*, Left subclavian artery.

**Table E4:** Comparison of operative details for patients with transient postoperative stroke (n = 29), those without stroke (n = 3643), and those with persistent postoperative stroke (n = 93)

Variable	No stroke (n = 3643)	Transient stroke (n = 29)	*P*	Transient stroke (n = 29)	Persistent stroke (n = 93)	*P*
Elective repair	2762 (75.8)	20 (69.0)	.4	20 (69.0)	74 (79.6)	.2
Crawford extent of repair						
Extent I	956 (26.2)	14 (48.3)	**.007**	14 (48.3)	25 (26.9)	**.03**
Extent II	1203 (33.0)	12 (41.4)	.3	12 (41.4)	50 (53.8)	.2
Extent III	740 (20.3)	3 (10.3)	.2	3 (10.3)	8 (8.6)	.8
Extent IV	744 (20.4)	0	**.006**	0	10 (10.8)	.07
Aortic repair details						
Clamping proximal to LSCA	566 (15.5)	11 (37.9)	**<.001**	11 (37.9)	18 (19.4)	**.04**
Distal hemiarch or other arch repair (concomitant)	26 (0.7)	0	.6	0	1 (1.1)	.6
LSCA bypass at time of repair	66 (1.8)	2 (6.9)	**.04**	2 (6.9)	6 (6.5)	.9
ET/FET completion anastomosis	180 (4.9)	2 (6.9)	.6	2 (6.9)	9 (9.7)	.6
Chronic untreated arch dissection	256 (7.0)	3 (10.3)	.5	3 (10.3)	7 (7.5)	.6
Segmental artery reattachment	1842 (50.6)	21 (72.4)	**.02**	21 (72.4)	62 (66.7)	.6
Aortic clamp time, min	51 [38-67] (n = 3624)	64 [47-77] (n = 28)	**.03**	60.0 ± 21.8 (n = 28)	59.2 ± 20.0 (n = 91)	.5
Management of visceral or renal arteries						
Endarterectomy, stenting, or bypass grafting of visceral/renal arteries (any)	1601 (43.9)	6 (20.7)	**.01**	6 (20.7)	41 (44.1)	**.02**
Bypass grafting	1042 (28.6)	4 (13.8)	.08	4 (13.8)	32 (34.4)	**.03**
Endarterectomy	941 (25.8)	2 (6.9)	**.02**	2 (6.9)	24 (25.8)	**.03**
Stenting	301 (8.3)	2 (6.9)	.8	2 (6.9)	12 (12.9)	.4
Adjuncts						
Cerebrospinal fluid drainage	1755 (48.2)	21 (72.4)	**.009**	21 (72.4)	62 (66.7)	.6
Left heart bypass	1637 (44.9)	21 (72.4)	**.003**	21 (72.4)	57 (61.3)	.3
Hypothermic circulatory arrest	62 (1.7)	1 (3.4)	.5	1 (3.4)	3 (3.2)	>.9
Cold renal perfusion	2177 (59.8)	14 (48.3)	.2	14 (48.3)	49 (52.7)	.7
Selective visceral perfusion	894 (24.5)	12 (41.4)	**.04**	12 (41.4)	36 (38.7)	.8

All patients with persistent stroke were excluded from this analysis. Values in bold indicate *P* < .05. *LSCA*, Left subclavian artery; *ET*, elephant trunk; *FET*, frozen elephant trunk.

**Table E5:** Comparison of select early outcomes for patients with transient postoperative stroke (n = 29), those without stroke (n = 3643), and those with persistent postoperative stroke (n = 93)

Variable	No stroke (n = 3643)	Transient stroke (n = 29)	*P*	Transient stroke (n = 29)	Persistent stroke (n = 93)	*P*
Operative death	267 (7.3)	1 (3.4)	.4	1 (3.4)	57 (61.3)	**<.001**
30-d death	166 (4.6)	0	.2	0	45 (48.4)	**<.001**
Ischemic stroke	0	24 (82.8)	–	24 (82.8)	71 (76.3)	.5
Hemorrhagic stroke	0	5 (17.2)	–	5 (17.2)	26 (28.0)	.2
Persistent paraplegia	99 (2.7)	0	.4	0	11 (11.8)	.05
Rescue CSFD	45 (1.2)	1 (3.4)	.3	1 (3.4)	1 (1.1)	.4
New-onset atrial fibrillation	570 (15.6)	5 (17.2)	.8	5 (17.2)	25 (26.9)	.3
Respiratory failure necessitating tracheostomy	303 (8.3)	10 (34.5)	**<.001**	10 (34.5)	29 (31.2)	.7
Discharge with stroke	0	0	–	0	36 (38.7)	**<.001**

Values are n (%). All patients with persistent stroke were excluded from this analysis. Values in bold indicate *P* < .05. *CSFD*, Cerebrospinal fluid drainage.

**Table E6:** Causes of operative death in patients (n = 40) with persistent ischemic stroke

Age, y	Sex	CKD	Repair	POD	Cause of death (comment)
54	F	Y	TAAA - I	1	Ischemic stroke
56	M	N	TAAA - I	1	Intraoperative cardiac arrest and coagulopathy leading to anoxic brain injury
10	M	N	TAAA - II	4	Ischemic and hemorrhagic strokes in young patient recovering from stroke and paraplegia related to previous aortic repair
65	M	N	TAAA - I	4	Ischemic stroke
67	M	Y	TAAA - II	4	Brain embolism
75	M	Y	TAAA - I	4	Cardiac tamponade leading to ischemic stroke
78	M	Y	TAAA - II	4	MSOF secondary to myocardial infarction (induced by heart failure and cerebral infarction)
70	F	Y	TAAA - II	5	Ischemic stroke and MSOF
81	F	N	TAAA - II	5	Ischemic stroke progressing to MSOF
64	M	Y	TAAA - II	6	Ischemic stroke
77	M	N	TAAA - I	6	Sudden respiratory arrest leading to anoxic brain injury
69	M	Y	TAAA - I	7	Sudden cardiac arrest and anoxic brain injury
72	M	Y	TAAA - II	7	Coagulopathy and cardiac failure leading to MSOF with irreversible brain injury
81	M	Y	TAAA - II	7	Ischemic stroke
83	F	Y	TAAA - II	7	Ischemic stroke
62	M	Y	TAAA - II	8	Ischemic stroke
71	F	N	TAAA - I	10	Ischemic stroke
76	F	Y	TAAA - II	10	Cardiac and respiratory failure leading to ischemic stroke and MSOF
60	M	Y	TAAA - II	11	Cardiopulmonary failure leading to MSOF and ischemic stroke
71	M	Y	TAAA - IV	13	Ischemic stroke leading to MSOF
71	M	Y	TAAA - IV	14	Bowel ischemia and ischemic stroke with MSOF and sepsis
85	M	Y	TAAA - II	14	MSOF
40	M	N	TAAA - II	15	MSOF
81	F	N	TAAA - III	15	Pneumonia with respiratory failure and sepsis precipitating MSOF
76	M	Y	TAAA - IV	16	Sepsis, ischemic stroke, and MSOF
62	M	N	TAAA - III	20	Coagulopathy and aspiration leading to ischemic stroke
78	M	N	TAAA - I	20	Bowel ischemia, sepsis, and MSOF
67	F	N	TAAA - II	24	Ischemic stroke
43	M	N	TAAA - IV	26	Cardiac failure and ischemic stroke leading to MSOF
74	M	Y	TAAA - II	26	Ischemic stroke
58	M	N	TAAA - I	30	Cardiac failure leading to anoxic brain injury and sepsis
75	F	Y	TAAA - II	38	Unknown; ischemic stroke occurred on POD 4
81	F	Y	TAAA - II	38	Pulmonary failure and pneumonia leading to sepsis and MSOF
83	M	Y	TAAA - III	39	Ischemic stroke, ischemic bowel, and MSOF
76	M	N	TAAA - II	40	Respiratory failure with ARDS, MSOF leading to cardiac arrest and anoxic brain injury
72	M	N	TAAA - I	46	Respiratory failure with ARDS leading to MSOF and sepsis
78	M	N	TAAA - II	59	Embolic stroke, respiratory failure, pneumonia, and sepsis
74	M	Y	TAAA - I	71	Respiratory failure and sepsis
74	F	Y	TAAA - III	81	Bronchopleural fistula and empyema with MSOF and sepsis
85	M	N	TAAA - II	86	Ischemic stroke and bradyarrhythmia

*CKD*, Chronic kidney disease; *POD*, postoperative day; *F*, female; *Y*, yes; *TAAA*, thoracoabdominal aortic aneurysm; *M*, male; *N*, no; *MSOF*, multisystem organ failure; *ARDS*, acute respiratory distress syndrome.

**Table E7:** Causes of operative death in patients (n = 17) with persistent hemorrhagic stroke

Age, y	Sex	CSFD	Repair	POD	Cause of death (comment)
42	M	Intraoperative	TAAA - II	2	Hemorrhagic stroke
45	F	Intraoperative	TAAA - II	2	Ruptured intracranial aneurysm leading to subarachnoid hemorrhage and stroke
55	M	Intraoperative	TAAA - II	2	Hemorrhagic stroke
71	F	Intraoperative	TAAA - I	2	Anoxic brain injury
37	F	Intraoperative	TAAA - II	3	Hemorrhagic stroke
75	M	Intraoperative	TAAA - II	3	Cerebellar hemorrhage
48	M	Intraoperative	TAAA - II	5	Respiratory failure and hemorrhagic stroke
37	M	Intraoperative	TAAA - II	7	Cardiopulmonary failure, subarachnoid hemorrhage, and sepsis
66	M	No	TAAA - I	8	Hemorrhagic stroke
66	F	No	TAAA - II	8	Myocardial infarction leading to MSOF
60	M	Intraoperative	TAAA - II	9	Respiratory failure leading to MSOF leading to hemorrhagic stroke
79	F	Intraoperative	TAAA - IV	11	Hemorrhagic stroke
81	M	Intraoperative	TAAA - II	20	MSOF and sepsis
76	M	Rescue	TAAA - IV	21	Respiratory and renal failure leading to MSOF
48	M	Intraoperative	TAAA - II	52	Hemorrhagic stroke leading to MSOF
56	F	Intraoperative	TAAA - II	81	Intraparenchymal hemorrhage (stroke)
80	M	Intraoperative	TAAA - I	107	Respiratory failure and stroke progressing to MSOF

*CSFD*, Cerebrospinal fluid drainage; *POD*, postoperative day; *M*, male; *TAAA*, thoracoabdominal aortic aneurysm; *F*, female; *MSOF*, multisystem organ failure.

**Table E8:** Univariate analysis for the logistic regression model for persistent ischemic stroke

Characteristics	Odds ratio	*P*
Age, increasing by year	1.04	**.001**
Heritable thoracic aortic disease	0.45	.06
Diabetes	2.05	**.03**
Cerebrovascular disease	2.01	**.008**
Previous stroke	2.18	**.01**
Chronic kidney disease	2.52	**<.001**
Body mass index, increasing by kg/m^2^	1.04	.08
Symptomatic	1.80	**.04**
Peripheral vascular disease	1.78	**.02**
Crawford extent II repair	2.06	**.003**
Crawford extent III repair	0.50	.07
Crawford extent IV repair	0.50	.07
Segmental artery reattachment	1.57	.07
Aortic clamp time, increasing by min	1.01	.06
Stenting of visceral and renal arteries	2.04	**.03**
Left heart bypass	1.65	**.04**
Cerebrospinal fluid drainage	1.53	.08
New-onset atrial fibrillation	2.11	**.005**

All variables in Tables 1 and 2 were considered, as was new-onset atrial fibrillation in Table 3, and all variables included in the table were significant to an alpha of 0.1 on univariate analysis. Values in bold indicate *P* < .05.

**Table E9:** Univariate analysis for the logistic regression model for persistent and transient ischemic stroke

Characteristics	Odds ratio	*P*
Age, increasing by year	1.04	**<.001**
Heritable thoracic aortic disease	0.38	**.02**
Diabetes	1.77	.06
Cerebrovascular disease	2.01	**.002**
Prior stroke	1.80	**.04**
Chronic kidney disease	2.59	**<.001**
Symptomatic	1.89	**.01**
Peripheral vascular disease	1.51	.07
Crawford extent II repair	1.89	**.002**
Crawford extent III repair	0.47	**.02**
Crawford extent IV repair	0.36	**.006**
Segmental artery reattachment	1.74	**.01**
Aortic clamp time, increasing by min	1.01	**.02**
Stenting of visceral and renal arteries	1.77	.06
Left heart bypass	1.90	**.002**
Selective perfusion of visceral arteries	1.62	**.03**
Cerebrospinal fluid drainage	1.82	**.005**
Clamping proximal to LSCA	1.73	**.02**
New-onset atrial fibrillation	1.93	**.006**

All variables in Tables 1 and 2 were considered, as was new-onset atrial fibrillation in Table 3, and all variables included in the table were significant to an alpha of 0.1 on univariate analysis. Values in bold indicate *P* < .05. *LSCA*, Left subclavian artery.

**Table E10:** Univariate analysis for the logistic regression model for persistent hemorrhagic stroke

Characteristics	Odds ratio	*P*
Age, increasing by year	0.97	**.03**
Heritable thoracic aortic disease	3.30	**.004**
Aortic dissection	2.55	**.02**
Prior myocardial infarction	0.18	.09
COPD	0.45	.08
Crawford extent II repair	4.24	**<.001**
Segmental artery reattachment	3.85	**.007**
Aortic clamp time, increasing by min	1.02	**.003**
Endarterectomy of visceral and renal arteries	0.25	.06
Left heart bypass	3.82	**.004**
Cerebrospinal fluid drainage	12.20	**<.001**

All variables in Tables 1 and 2 were considered, and all variables included in the table were significant to an alpha of 0.1 on univariate analysis. Values in bold indicate *P* < .05. *COPD*, Chronic obstructive pulmonary disease.

**Table E11:** Univariate analysis for the logistic regression model for persistent and transient hemorrhagic stroke

Characteristics	Odds ratio	*P*
Age, increasing by year	0.97	**.02**
Heritable thoracic aortic disease	2.86	**.006**
Aortic dissection	2.22	**.03**
Crawford extent II repair	3.45	**.001**
Crawford extent III repair	0.14	.051
Crawford extent IV repair	0.28	.09
Segmental artery reattachment	3.86	**.003**
Aortic clamp time, increasing by min	1.02	**.003**
Endarterectomy of visceral and renal arteries	0.32	.06
Left heart bypass	3.96	**.001**
Cold renal perfusion	0.52	.08
Selective perfusion of visceral arteries	3.97	**<.001**
Cerebrospinal fluid drainage	6.90	**<.001**

All variables in Tables 1 and 2 were considered, and all variables included in the table were significant to an alpha of 0.1 on univariate analysis. Values in bold indicate *P* < .05.

**Table E12:** Description of imputed data used in logistic regression models

Variable	No stroke (n = 3643)	Transient stroke (n = 29)	Persistent stroke: Ischemic (n = 71)	Persistent stroke: Hemorrhagic (n = 22)	*P*
Maximum distal aortic diameter, cm	6.2 [5.5-7.2]	5.8 [5.2-6.4]	6.3 [5.7-7.0]	5.8 [5.2-6.4]	.2
Max distal aortic diameter, cm (imputed, n = 428)	6.3 [5.6-7.0]	5.8 [5.5-6.6]	6.4 [5.7-7.0]	5.8 [5.2-6.5]	.3
Body mass index, kg/m^2^	25.8 [22.8-29.1]	27.3 [22.9-31.5]	27.0 [22.8-29.2]	27.3 [22.9-31.5]	.1
Body mass index, kg/m^2^ (imputed, n = 482)	25.8 [23.1-28.7]	26.2 [23.9-27.9]	26.5 [24.2-29.8]	27.3 [24.4-30.7]	.3
Chronic kidney disease∗	1395 (38.3)	16 (55.2)	42 (59.2)	10 (45.5)	**.001**
Chronic kidney disease∗ (imputed, n = 297)	1531 (42.0)	18 (62.1)	46 (64.8)	11 (50.0)	**<.001**
Aortic clamp time, min	51 [38-67]	64 [47-77]	57 [44-71]	64 [47-77]	**.02**
Aortic clamp time, min (imputed, n = 21)	51 [38-67]	60 [42-79]	57 [45-72]	63 [48-77]	**.01**

Values are n (%) or median [quartile 1-quartile 3]. Values in bold indicate *P* < .05.

∗ Preoperative estimated glomerular filtration rate <60 mL/min/1.73 m^2^.

**Table E13:** Univariate and multivariate analysis for the logistic regression model for operative death in patients with a persistent stroke (n = 93)

Characteristics	Univariate analysis		Multivariable analysis	
	Odds ratio	*P*	Odds ratio	*P*
Preoperative atrial fibrillation	4.07	.08	–	–
Aortic dissection	2.19	.08	–	–
Previous aortic repair	2.54	.1	–	–
Aortic clamp time, increasing by min	1.03	**.03**	1.03	**.03**

All variables in Tables 1 and 2 were considered, and all variables included in the table were significant to an alpha of 0.1 on univariate analysis. Values in bold indicate *P* < .05.
